# Sex differences in depressive symptoms and tolerability after treatment with selective serotonin reuptake inhibitor antidepressants: Secondary analyses of the GENPOD trial

**DOI:** 10.1177/0269881120986417

**Published:** 2021-02-26

**Authors:** Marilia Gougoulaki, Glyn Lewis, David J Nutt, Tim J Peters, Nicola J Wiles, Gemma Lewis

**Affiliations:** 1 Barnet, Enfield and Haringey Mental Health NHS Trust, London, UK; 2 Division of Psychiatry, Faculty of Brain Sciences, University College London, London, UK; 3 Department of Psychiatry, Division of Brain Science, Imperial College, London, UK; 4 Bristol Medical School, University of Bristol, Bristol, UK; 5 Centre for Academic Mental Health, Population Health Sciences, Bristol Medical School, University of Bristol, Bristol, UK

**Keywords:** Antidepressants, sex differences, depression, GENPOD

## Abstract

**Background:**

Differences in serotonergic neurotransmission could lead to sex differences in depressive symptoms and tolerability after treatment with selective serotonin reuptake inhibitors (SSRIs).

**Aims:**

We investigated whether women have greater reductions in depressive symptoms than men after treatment with an SSRI (citalopram) compared with a noradrenaline reuptake inhibitor (reboxetine) control, and after antidepressant treatment irrespective of class. We also investigated tolerability and the influence of menopausal status.

**Methods:**

Secondary analyses of the GENPOD (GENetic and clinical Predictors Of treatment response in Depression) trial. Six hundred and one people with depression were recruited from UK primary care and randomized to citalopram or reboxetine. Beck Depression Inventory (BDI-II) score at 6 weeks was the primary outcome. Secondary outcomes included BDI-II score at 12 weeks, and physical symptoms and treatment discontinuation. We calculated main effects and interaction terms using linear and logistic regression models.

**Results:**

There was no evidence that women experienced greater reductions in depressive symptoms than men when treated with citalopram compared with reboxetine. We also found no evidence of sex differences at six or 12 weeks (irrespective of antidepressant class): men scored −0.31 (95% confidence interval (CI) −2.23 to 1.62) BDI-II points lower than women at six weeks and −0.44 (95% CI −2.62 to 1.74) points lower at 12 weeks. There was no evidence of sex differences in physical symptoms or treatment discontinuation and no evidence for an influence of menopausal status.

**Conclusion:**

Citalopram was not more effective in women compared with men and there was no difference in tolerability. Women and men had similar prognosis after SSRI treatment and similar prognosis regardless of antidepressant class. Findings were unaltered by menopausal status.

## Introduction

Depression is a leading cause of disability worldwide and a major contributor to the global burden of disease (Mathers and Loncar, 2006). People with depression are usually managed in primary care and antidepressants are often the first-line treatment. However, many patients who receive an antidepressant do not experience a meaningful reduction in their depressive symptoms, and clinicians have little guidance on which patients are more likely to respond to the different types of antidepressants. Identifying patient groups more likely to respond to certain antidepressants would improve the management of depression in primary care and other clinical settings.

Selective serotonin reuptake inhibitors (SSRIs) are the most commonly prescribed class of antidepressants. SSRIs bind to the serotonin reuptake transporter on presynaptic serotonin terminals and prevent the reuptake of serotonin. This leads to increased serotonin levels in the synapse. There is evidence of differences between the serotonergic neurotransmitter systems of women and men (Labaka et al., 2018; Ngun et al., 2011), although findings are inconsistent (Munro et al., 2012). Oestrogen, the primary female sex hormone, organizes and activates brain neurocircuitry and modulates the expression of serotonin receptors and transporters (Chavez et al., 2010). Oestrogen induces the expression of neuropeptides implicated in mood variation, with evidence that oestrogen-dependent neuropeptide function is associated with differences in response to antidepressant medication (Unschuld et al., 2010). Acute tryptophan depletion has been shown to lower mood in healthy females but not males (Bell et al., 2001; Ellenbogen et al., 1996). There is also evidence that men have a higher rate of synthesis of serotonin than women (Nishizawa et al., 1997). This may increase baseline serotonin availability in men compared with women, potentially blunting the pharmacological effects of SSRIs in men. It is therefore possible that women may respond better to SSRI antidepressants than men, and women may respond better to SSRIs than to other antidepressants (whereas for men there may be no difference between SSRIs and other antidepressants) (Bigos et al., 2009).

Evidence on whether there are greater reductions in depressive symptoms among women than men after treatment with SSRIs is inconsistent and existing studies have several limitations. In the large STAR*D study, remission of depression was higher in women than men receiving citalopram (Young et al., 2009). However, this finding was observed on one depression measure (the Hamilton Rating Scale for Depression (HRSD)) but not another (Quick Inventory of Depression Symptomatology), leading to doubts about reliability as there is evidence of high concordance between the two measures. The discrepancy could also be due to the method of administration of the HRSD in STAR*D – through telephone interviews and without clinical observation. Another small observational study (*N*=66) of fluvoxamine found that women had fewer depressive symptoms than men at six week follow-up (Naito et al., 2007). In the large CRESCEND cohort study of antidepressants in primary care, women had higher 12-week remission rates than men (Kim et al., 2011). This study included SSRIs, along with other classes of antidepressant, but did not adjust for or compare outcomes between women and men on different antidepressants. Other observational studies found no differences in response to SSRIs between women and men (Parker et al., 2003; Pinto-Meza et al., 2006; Thiels et al., 2005).

A limitation of these observational studies is that, with the exception of the CRESCEND study (Kim et al., 2011), all participants were taking the same antidepressant. The CRESCEND study included SSRIs, newer antidepressants (e.g. venlafaxine and mirtazapine) and older antidepressants (e.g. amitriptyline and nortriptyline). However, there was no comparison of outcomes between women and men taking the different classes of antidepressant. It is therefore uncertain whether greater reductions in depressive symptoms among women were due to specific effects of the SSRI, or whether women were generally recovering faster than men after antidepressant treatment.

Several randomized controlled trials have compared SSRIs with tricyclic or serotonin–norepinephrine reuptake inhibitor antidepressants and found greater reductions in depressive symptoms among women treated with SSRIs than men (Berlanga and Flores-Ramos, 2006; Haykal and Akiskal, 1999; Hildebrandt et al., 2003; Joyce et al., 2002, 2003; Khan et al., 2005; Kornstein et al., 2014; Sramek et al., 2016). However, in several other randomized controlled trials (RCTs), there was no evidence that women responded better to SSRIs than men, and no evidence that women responded better to SSRIs than to other antidepressants (Khan et al., 2005; Pinto-Meza et al., 2006). Findings from larger trials tend to support the hypothesis that women and men do not respond differently to SSRIs (Berlanga and Flores-Ramos, 2006; Hildebrandt et al., 2003; Martényi et al., 2001).

Of the existing observational studies and RCTs, many were small and may have lacked statistical power to investigate sex differences (Haykal and Akiskal, 1999; Hildebrandt et al., 2003; Martényi et al., 2001; Naito et al., 2007), which require interactions and large samples in order to produce valid findings. Studies that are under-powered can increase the risk of Type I (false positive) as well as Type II (false negative) errors (Button et al., 2013). Among the studies comparing different antidepressants, an important limitation is that the various drugs studied, while having some pharmacological differences, all inhibit the reuptake of serotonin, making any comparison of differential effectiveness among women and men difficult.

Noradrenaline reuptake inhibitors (NaRIs) inhibit the reuptake of noradrenaline from the synapse back into the presynaptic terminal. NaRIs are a useful comparator with SSRIs in research studies because they affect a different neurotransmitter system (noradrenaline). To test the hypothesis that men and women respond differently to SSRIs because of differences in how serotonin is processed, we need to compare antidepressants which target different neurotransmitter systems. NaRIs are therefore a useful comparator to SSRIs for this hypothesis.

To our knowledge, only one small RCT (*N*=86) has compared response to SSRIs with NaRIs among men and women (Berlanga and Flores-Ramos, 2006). After treatment with citalopram (SSRI), women showed greater reductions in depressive symptoms than men. Women treated with citalopram also had greater reductions in depressive symptoms than women treated with reboxetine (NaRI). In men, no differences were observed.

Menopausal status and the perimenopausal period, in particular, has been linked to prevalence and relapse of depression (Freeman et al., 2004). There is contradictory evidence on the effect of menopausal status on response to antidepressants in women. Some studies have found that SSRIs are more effective in pre- than in postmenopausal women (Pae et al., 2009; Pinto-Meza et al., 2006). These studies were limited by small sample sizes and often did not adjust for other factors which may have affected results (such as previous history of depression). A secondary analysis of the STAR*D trial found no evidence of an effect of menopausal status on response to citalopram (Kornstein et al., 2013). This analysis was limited by lack of a control or comparator agent, as all included women received treatment with citalopram only.

Few studies have explored sex differences in tolerability of antidepressant treatments. One study pooled data from several double-blind, placebo controlled clinical trials and found equivalent adverse event profiles for men and women taking duloxetine (Stewart et al., 2006). There was some evidence of sex differences in rates of treatment discontinuation, with more men discontinuing (18.6% *vs*. 13.5% of women). An earlier study found decreased rates of treatment discontinuation in women taking sertraline (a SSRI) versus imipramine (a tricyclic antidepressant) (Kornstein et al., 2000). The study recruited only individuals with chronic depression (major depression with an established diagnosis of dysthymia) and it is unclear whether these can be generalized to all patients with depression.

In this study, we report secondary analyses of the GENPOD (GENetic and clinical Predictors Of treatment response in Depression) trial. We tested the hypothesis that women would have greater reductions in depressive symptoms than men after treatment with an SSRI compared with a NaRI control. We also investigated whether women had a better depression severity prognosis after antidepressant treatment, regardless of antidepressant class. We also investigated sex differences in antidepressant tolerability. We then tested whether the effects of sex on depression symptom severity and tolerability after treatment with antidepressants were influenced by menopausal status.

## Methods

The full protocol for the GENPOD trial has been published elsewhere (Thomas et al., 2008). In brief, the study was a multi-centre RCT of 601 patients aged 18–74 years, recruited from primary care surgeries in Bristol, Birmingham and Newcastle, UK. Patients were referred by General Practitioners (GPs) and were randomly allocated to a NaRI (reboxetine 4 mg twice daily) or a SSRI (citalopram 20 mg daily). Eligibility criteria were that patients met ICD-10 criteria for a depressive episode (ICD-10 code F32), assessed using the computerized Clinical Interview Schedule–Revised (CIS-R) (Lewis et al., 1992). The CIS-R is a computerized, self-administered, fully structured interview measuring 14 common mental disorder symptom groups. The CIS-R generates diagnoses meeting ICD-10 criteria for depressive or anxiety episodes, a total common mental disorders score, and a depression severity score (available range 0–21) created by the sum of the following five symptoms: depression, depressive ideas, fatigue, concentration, and sleep problems. Patients also had to score 15 or more on the Beck Depression Inventory (BDI-II) (Beck et al., 1997). We excluded patients unable to complete self-administered questionnaires, and those who had taken antidepressants in the last two weeks. Contraindications such as diagnoses of bipolar disorder, major drug or alcohol misuse disorders, and current psychosis were also excluded.

Ethical approval was obtained from the South West Research Ethics Committee (MREC 02/6/076) and research governance approval from Bristol, Manchester and Newcastle Primary Care NHS Trusts (Trial registration: ISRCTN31345163 and EudraCT number: 2004-001434-16).

### Randomization

Following the baseline assessment, eligible participants were asked to give written informed consent. Randomization was performed with a computer-generated code, administered centrally and communicated by telephone, concealed in advance from the researcher. Allocation was stratified by severity of overall symptoms (CIS-R total score <28 or >28) and centre, using variable block sizes to maximize concealment. The researcher gave the allocated medication to the participant. Neither researchers nor participants were masked to allocated treatment (due to difference in the treatment regimens and because double placebos were infeasible).

### Allocated treatments

Participants randomized to citalopram were prescribed 20 mg daily, which has been shown to be sufficient dosage for treatment (Furukawa et al., 2019). Those allocated to reboxetine were advised to start on 2 mg twice daily and increase this to 4 mg twice daily after 4 days. Acute doses of 4 mg of reboxetine have been found to increase cortisol levels, indicative of increased noradrenergic function (Schüle et al., 2004). All participants were advised to contact their GP if they wished to increase the dose of their medication.

### Outcome measures

Outcome data were recorded six and 12 weeks after randomization. The primary outcome was total score on the BDI-II, a self-report 21-item scale that assesses the severity of depressive symptoms in the past two weeks. Scores range from 0 to 63, with higher scores indicating more severe symptoms. As a secondary outcome we analysed BD-II scores at 12 weeks. Self-administered outcomes were used because of the potential for bias from clinician-administered measures in a non-masked trial such as this.

### Adherence

Adherence was assessed at two, six and 12 weeks using self-report and a pill count of returned medication. Participants were asked about their use of antidepressant medication in the follow-up questionnaires (six closed response options: I have not taken any of my tablets; I have taken hardly any of my tablets; I have taken less than half of my tablets; I have taken more than half of my tablets; I have taken nearly all my tablets; I have taken my tablets every day). Adherence responses were stratified in two categories: participants who had ‘taken medications daily or nearly daily’ or ‘taken less than nearly all’. Those who had taken their medication daily or nearly daily were classed as adherent, whereas those who had taken less than nearly all their medication were classed as non-adherent.

### Physical symptoms and treatment discontinuation

Treatment discontinuation was measured at six and 12 weeks of follow-up by asking patients whether they had decided to stop taking their allocated antidepressant treatment. Physical symptoms that could be antidepressant side effects were recorded at baseline (medication-free) and at six and 12 weeks after randomization using a modified version of the Toronto Side Effects Scale (14 symptoms) (Crawford et al., 2014). Individuals reported the number of days that they had experienced each physical symptom in the last week (0, 1–3, 4–7 days). We generated a total score for the number of physical symptoms reported by each participant.

### Justification of the sample size

Details of the sample size calculations and the impact of final recruitment figures on power were given in the protocol paper (Thomas et al., 2008). Sample size was primarily driven by the genetic hypothesis in the primary analysis (that patients with depression and the l/l genotype of 5-HTTLPR would show a better response to the SSRI citalopram than to the NaRI reboxetine) (Lewis et al., 2011), with a revised target of 570 participants for the primary analysis. GENPOD was adequately powered to address the secondary question of differential response to treatment based on severity of depression assessed with a binary variable (that is, the interaction between severity and treatment allocation) (Wiles et al., 2012). Estimating statistical power is complicated when interaction tests are involved, but there is no reason to propose a substantially different target magnitude for the interaction involved in this secondary analysis compared with the original, and we can therefore reasonably contend sufficient power for our secondary analyses of the effects of sex. Moreover, the power for the main effect of sex (our second research question) will be substantially higher than for the interaction, as the confidence intervals will demonstrate.

## Statistical analyses

All analyses were conducted using STATA version 13.

### Comparing reductions in depressive symptoms at follow-up among men and women taking citalopram or reboxetine

We used a linear regression with BDI-II scores at six weeks as a continuous outcome and treatment allocation and sex as the main exposures. The model was adjusted for baseline BDI-II scores, centre and the CIS-R total score (stratification variable). To investigate the hypothesis of a differential response to citalopram versus reboxetine amongst women compared with men, we included an interaction between treatment (citalopram/reboxetine) and sex in this model and reported the interaction coefficient and the *p*-value accompanying this coefficient. We then repeated this analysis with BDI-II scores at 12 weeks as a secondary outcome.

### Comparing reductions in depressive symptoms at follow-up among men and women, irrespective of antidepressant class

We generated a linear regression with BDI-II scores at six weeks as the outcome and sex as the exposure. We then adjusted for treatment allocation, centre and the CIS-R total score (stratification variable; Model 1). We further adjusted for baseline BDI-II scores (Model 2) and then for factors differing between men and women that were related to the outcome: age, employment status, history of depression, and previous treatment for depression (Model 3). We repeated this analysis with BDI-II scores at 12 weeks as a secondary outcome.

### Comparing tolerability among men and women taking citalopram or reboxetine

We used a linear regression with physical symptoms at six weeks as a continuous outcome and treatment allocation and sex as the main exposures. The model was adjusted for baseline physical symptom score, centre and the CIS-R total score (stratification variable). To investigate the hypothesis of differential tolerability of citalopram versus reboxetine amongst women compared with men, we included an interaction between treatment (citalopram/reboxetine) and sex in this model and reported the interaction coefficient and the *p*-value accompanying this coefficient. We repeated this analysis with physical symptom scores at 12 weeks as a secondary outcome. Similar models were run using logistic regression for the treatment discontinuation outcome at six and 12 weeks.

### Comparing effect of sex on reductions in depressive symptoms at follow-up among premenopausal and perimenopausal women compared with men

We stratified participants into two age groups (under the age of 45 and 45 years old and older). We used age 45 as an age cut-off below which women were likely to be premenopausal (Santoro et al., 2015). We repeated analyses of reductions in depressive symptoms at follow-up, adjusting for participant age as well the new binary age variable. We then added a three-way interaction between treatment, sex and the binary age variable.

### Sensitivity analyses

We repeated analyses on depression severity after treatment with citalopram and reboxetine among men and women using BDI-II scores from waves 6 and 12 as a repeated measures outcome analysed within a linear multilevel regression model. BDI-II scores at six and 12 weeks were clustered within individuals, and we included a random intercept at the level of the individual. We repeated analyses of the primary outcome, including only those who had reported taking their medication for at least four weeks (treatment adherence). We also repeated these analyses using the Hospital Anxiety and Depression Scale (HADS) at six and 12 weeks as the outcome.

We repeated analyses on physical symptoms and treatment discontinuation by using scores from waves 6 and 12 as a repeated measures outcome.

We examined the impact of menopausal status on antidepressant tolerability after treatment with citalopram and reboxetine. We used linear regression with physical symptom score at six and 12 weeks and included an interaction between treatment (citalopram/reboxetine) and sex. We restricted this analysis to participants under the age of 45 years as a proxy for premenopausal status. We repeated analyses at six and 12 weeks, restricting our participants to those at and over the age of 45 years, as a proxy for perimenopausal or postmenopausal status. Similar models were run using logistic regression for the impact of menopausal status on treatment discontinuation outcome at six and 12 weeks.

## Results

The CONSORT flow chart and baseline comparability of randomized groups are published elsewhere (Lewis et al., 2011). Between October 2005 and February 2006, 601 participants (408 women and 193 men) were randomized either to citalopram (*n*=298) or to reboxetine (*n*=303). The mean age of the participants was 38.8 years (SD=12.4) and 68% (*n*=408) were female. Ninety-two per cent had moderate (*n*=305) or severe (*n*=245) depression according to ICD-10 criteria. The six week follow-up assessment was done by 91% of participants and retention did not differ by study arm (citalopram, *n*=274; reboxetine, *n*=272; *p*=0.83). At 12 weeks, 81% of those randomized were retained, and retention did not differ by study arm (citalopram, *n*=253; reboxetine, *n*=233; *p*=0.72).

At six weeks, adherence was higher among those randomized to citalopram (*n*=246, 83%, on medication for citalopram and *n*=193, 64% on medication for reboxetine). Of 98 men randomized to citalopram, 16 had discontinued treatment by six weeks (16.3%). Of 95 men randomized to reboxetine, 37 had discontinued treatment at six weeks (38.9%).Of 208 women randomized to reboxetine, 71 had discontinued treatment at six weeks (34.1%). Of 200 women randomized to citalopram, 25 had discontinued treatment at six weeks.

GPs increased the dose of citalopram for 55 (20%) participants in total, from 20 mg to 30 mg (*n*=11), 40 mg (*n*=33), and 60 mg (*n*=11). A smaller number of those randomized to reboxetine had their dose increased, from 4 mg twice daily to 10 mg (*n*=3), 12 mg (*n*=9), 16 mg (*n*=1).

### Baseline comparability of women and men

Baseline characteristics of women and men are shown in Table 1. The mean baseline BDI-II score was 34.7 (SD 9.6) for women and 31.4 (SD 9.4) for men. Compared with women, men were older and more likely to be employed full-time. Women were more likely to have a history of depression and to have used antidepressants before.

**Table 1.** Baseline characteristics of the sample according to sex.

Characteristic	Women *n*=408	Men *n*=193	*p* value^a^
Age	37.8 (12.3)	41.1 (12.3)	0.0023
BDI-II score; possible range 0–63	34.7 (9.6)	31.4 (9.4)	0.0001
CIS-R total score; possible range 0–64	30.6 (8.1)	31.4 (7.7)	0.25
Number of life events	1.6 (1.3)	1.8 (1.4)^b^	0.11
Social support score; possible range 1–24	12.0 (3.7)	12.0 (4.0)^b^	0.98
Ethnicity			0.48
White	392 (96.1%)	183 (95.0%)	
Ethnic minority	16 (3.9%)	10 (5.2%)	
Employment status			<0.0001
Working full-time	127 (31.1%)	116 (60.1%)	
Working part-time	104 (25.5%)	10 (5.2%)	
Not in employment	177 (43.4%)	67 (34.7%)	
Marital status			0.74
Married or living as married	218 (53.4%)	98 (50.8%)	
Single	119 (29.2%)	56 (29.0%)	
Separated, divorced or widowed	71 (17.4%)	39 (20.2%)	
History of depression	312 (76.5%)	123 (64.1%)^b^	0.001
Previous antidepressants	245 (60.3%)^c^	80 (41.7%)	<0.0001
Suicidal ideation			0.29
None	16 (3.9%)	12 (6.2%)	
Hopelessness	89 (21.8%)	44 (22.8%)	
Life worthlessness	109 (26.7%)	38 (19.7%)	
Suicidal thoughts	146 (35.8%)	71 (36.8%)	
Suicidal plans	48 (11.8%)	28 (14.5%)	

Data are *n* (%) or mean (SD).

a *p*-values are from *t*-tests for continuous variables and chi-squared test for categorical variables.

b Missing data in one observation (*n*=192).

c Missing data in two observations (*n*=406).

BDI-II: Beck Depression Inventory; CIS-R: Clinical Interview Schedule – Revised

### Comparing reductions in depressive symptoms at follow-up among women and men taking citalopram or reboxetine

Differences in BDI-II scores at six and 12 weeks between women and men taking citalopram or reboxetine are shown in Table 2. At six weeks, there was no evidence that women had greater reductions in depressive symptoms than men after citalopram treatment, and no evidence that women responded better to citalopram than to reboxetine (interaction term between treatment allocation and sex: −1.13, 95% confidence interval (CI): −4.81 to 2.55, p=0.55). Results were similar for the secondary BDI-II outcome at 12 weeks (Table 2).

**Table 2.** Means and adjusted differences (and 95% CIs) in BDI-II scores at 6 and 12 weeks, according to citalopram and reboxetine groups in women and men.

Sex	Citalopram		Reboxetine		Adjusted difference (95% CI) between citalopram and reboxetine
	*n*	Mean (SD)	*n*	Mean (SD)	
Six weeks, *n*=546					
Women	185	19.5 (11.1)	188	20.3 (11.4)	1.52 (−0.63 to 3.66)
Men	89	17.6 (10.3)	84	17.9 (10.4)	0.37 (−2.52 to 3.26)
12 weeks, *n*=486					
Women	174	16.3 (12.2)	166	15.7 (11.5)	0.03 (−2.40 to 2.46)
Men	79	13.4 (9.5)	67	13.7 (10.6)	0.36 (−2.80 to 3.51)

Differences in means are calculated from linear regression models, higher scores indicating a worse outcome on reboxetine compared with citalopram (the reference category).

Differences in means were adjusted for the stratification variable (depression symptom severity <28 or ⩾28 on the CIS-R), centre and continuous baseline BDI-II scores.

BDI-II: Beck Depression Inventory; CI: confidence interval; CIS-R: Clinical Interview Schedule – Revised

### Comparing reductions in depressive symptoms at follow-up among men and women, irrespective of antidepressant class

In the univariable model, there was some evidence that women had more severe depressive symptoms than men at six weeks (difference in means −2.14, 95% CI −4.09 to −0.19, *p*=0.03; Table 3). However, after we adjusted for baseline BDI scores, there was no longer evidence of a difference in depressive symptoms between women and men (difference in means −0.37, 95% CI −2.25 to 1.52, *p*=0.67; Table 3). This was unchanged after further adjustment for variables that differed between women and men at baseline (age, history of depression, employment status, previous treatment for depression, and employment status; difference in means −0.15, 95% CI −2.09 to 1.79, *p*=0.55; Table 3). Findings were similar at 12 weeks (Table 3).

**Table 3.** Means and adjusted differences in means (95% CIs) in BDI-II scores at six and 12 weeks in women and men, irrespective of (and adjusted for) antidepressant.

Sex	Mean (SD) BDI-II scores	
	Six weeks	12 weeks
Women, *n*=373	19.9 (11.2)	16.0 (11.9)
Men, *n*=173	17.8 (10.3)	13.6 (10.0)
Model	Adjusted mean difference (95% CI) between women and men	
	Six weeks, *n*=546	12 weeks, *n*=486
Model 1^a^		
Women	Ref.	Ref.
Men	−2.16 (−4.10 to −0.20)	−2.52 (−4.71 to −0.34)
Model 2^b^		
Women	Ref.	Ref.
Men	−0.40 (−2.29 to 1.48)	−0.88 (−3.04 to 1.28)
Model 3^c^		
Women	Ref.	Ref.
Men	−0.60 (−2.53 to 1.35)	−0.82 (−2.99 to 1.37)

a Model 1: adjusted for treatment allocation, stratification variable (depression symptom severity <28 or ⩾28 on the CIS-R) and centre.

b Model 2: Model 1 adjusted for baseline BDI-II scores.

c Model 3: Model 2 adjusted for factors differing between women and men (age, employment status, history of depression and previous antidepressant treatment).

BDI-II: Beck Depression Inventory; CI: confidence interval; CIS-R: Clinical Interview Schedule – Revised; Ref.: reference

### Comparing tolerability among men and women taking citalopram or reboxetine

Differences in physical symptom scores at six and 12 weeks between women and men taking citalopram or reboxetine are shown in Table 5. At six weeks, there was no evidence of a difference (interaction term between treatment allocation and sex: −0.50, 95% CI: −3.01 to 2.00, *p*=0.69). Results were similar at 12 weeks (interaction term between treatment allocation and sex: −0.69, 95% CI: −3.10 to 1.71, *p*=0.57).

**Table 4.** Means and adjusted differences in mean (95% CIs) BDI-II scores at six and 12 weeks, according to citalopram and reboxetine groups in women and men who took their antidepressants for four weeks or more (adherence sensitivity analyses).

Sex	Citalopram		Reboxetine		Adjusted difference (95% CI) between citalopram and reboxetine
	*n*	Mean (SD)	*n*	Mean (SD)	
Six weeks, *n*=474					
Women	167	19.2 (11.2)	156	20.1 (11.6)	1.28 (−1.04 to 3.60)
Men	83	17.5 (9.8)	68	17.5 (9.8)	0.48 (−2.56 to 3.52)
12 weeks, *n*=384					
Women	153	15.8 (12.1)	117	15.1 (11.7)	−0.44 (−3.1 to 2.26)
Men	70	13.8 (9.5)	44	13.1 (9.9)	−0.18 (−3.83 to 3.45)

Differences in means are calculated from linear regression models, with higher scores indicating a worse outcome on reboxetine compared with citalopram (the reference category).

Differences in means are adjusted for the stratification variable (depression symptom severity <28 or ⩾28 on the CIS-R), centre and continuous baseline BDI-II scores.

BDI-II: Beck Depression Inventory; CI: confidence interval; CIS-R: Clinical Interview Schedule – Revised

**Table 5.** Means and adjusted differences (and 95% CIs) in physical symptom scores at six and 12-weeks, according to citalopram and reboxetine groups in women and men.

Sex	Citalopram		Reboxetine		Adjusted difference (95% CI) between citalopram and reboxetine
	*n*	Mean (SD)	*n*	Mean (SD)	
Six weeks, *n*=546					
Women	185	11.0 (8.0)	188	12.4 (7.8)	1.28 (−0.07 to 2.64)
Men	89	10.7 (7.9)	84	11.9 (9.1)	0.86 (−1.42 to 3.13)
12 weeks, *n*=486					
Women	174	9.9 (7.0)	166	11.3 (7.0)	0.83 (−0.48 to 2.15)
Men	79	9.9 (6.8)	67	10.3 (7.9)	0.11 (−1.98 to 2.2)

Differences in means are calculated from linear regression models, higher scores indicating a higher frequency of adverse events on reboxetine compared with citalopram (the reference category).

Differences in means were adjusted for the stratification variable (depression symptom severity <28 or ⩾28 on the CIS-R), centre and continuous baseline physical symptom scores.

CI: confidence interval; CIS-R: Clinical Interview Schedule – Revised

At six weeks, there was no evidence of a sex difference in treatment discontinuation, and no evidence that women were less likely to discontinue citalopram than reboxetine (odds ratio for interaction term between treatment allocation and sex: 1.35, 95% CI: 0.60 to 3.08, *p*=0.46). Results were similar at 12 weeks (odds ratio 1.18, 95% CI 0.58 to 2.41, *p*=0.65).

### Comparing effect of sex on reductions in depressive symptoms at follow-up among premenopausal and menopausal women compared with men

There was no evidence of a differential effect of sex on treatment response to citalopram and reboxetine at 6 weeks according to age as a proxy for menopausal status (interaction coefficient 5.04, 95% CI −2/67 to 12.74, *p*=0.20). Results were similar at 12 weeks (coefficient 1.39, 95% CI −7.35 to 10.14, *p*=0.75). Differences in BDI-II scores at six and 12 weeks between women likely to be premenopausal and postmenopausal and men taking citalopram or reboxetine are shown in Supplementary material Tables 1 and 2 online.

### Sensitivity analyses

In our repeated measures analyses using BDI scores at six and 12 weeks, there was no evidence of a difference in response to citalopram versus reboxetine in women compared with men (interaction term between treatment allocation and sex −0.13, 95% CI −3.57 to 3.29, *p*=0.94). There was no evidence, among women or men, that the treatment effect differed across time (the interaction term between treatment and time in women was 1.58, 95% CI −3.58 to 0.42, *p*=0.12 and in men: 0.45, 95% CI −2.30 to 3.19, *p*=0.75).

Restricting the analysis to patients who had taken their medication for a minimum of four weeks (Table 4), there was no evidence of a difference in response to citalopram versus reboxetine in women compared with men (the interaction term at the six-week follow-up was: −0.66, 95% CI −4.63 to 3.30, *p*=0.89 and at 12 weeks was: −0.20, 95% CI −5.00 to 4.20, *p*=0.94). Results were similar for depression severity prognosis (interaction term: −0.14, 95% CI: −2.28 to 2.01, *p*=0.90).

Using HADS depression scores as an outcome for our analysis, there was no evidence that women responded better to citalopram than reboxetine at six (interaction term between treatment allocation and sex: −0.40, 95% CI: −2.00 to 1.25, *p*=0.63) or 12 weeks (interaction term between treatment allocation and sex: 0.75, 95% CI: −1.11 to 2.62, *p*=0.43). Differences in HADS scores at six and 12 weeks between women and men taking citalopram or reboxetine are shown in Supplementary Table 3.

In repeated measures analyses of tolerability data from six- and 12-week follow-ups, there was no evidence of a difference in tolerability in response to citalopram versus reboxetine in women compared with men (interaction term between treatment allocation and sex 0.26, 95% CI −0.26 to 0.78, *p*=0.33). There was no evidence, among women or men, that physical symptom scores differed across time (the interaction term between physical symptom scores and time in women was −0.90, 95% CI −2.18 to 0.38, *p*=0.17 and in men: −1.27, 95% CI −2.85 to 0.31, *p*=0.11).

Similar effects were found in repeated measures analyses of treatment discontinuation at six- and 12- week follow-ups (interaction term between treatment allocation and sex, 0.91, 95% CI −0.68 to 1.23, *p*=0.58, and interaction term between treatment discontinuation and time in women −0.06, 95% CI −1.39 to 1.27, *p*=0.93 and in men: −0.35, 95% CI −2.36 to 1.66, *p*=0.73). Odds ratios for treatment discontinuation in men and women taking citalopram and reboxetine can be seen in Supplementary Table 4.

At six weeks, there was no evidence that premenopausal status affected sex differences in physical symptoms after treatment with citalopram and reboxetine (interaction term between treatment allocation and sex: −0.9, 95% CI: −4.07 to 2.27, *p*=0.58). Results were similar at 12 weeks (interaction term between treatment allocation and sex: −0.29, 95% CI: −3.42 to 2.85, *p*=0.86). Repeating the same analysis on participants at or over the age of 45 years as a proxy for perimenopausal and postmenopausal status yielded similar results at six and 12 weeks of treatment (interaction coefficient 1.02, 95% CI −3.17 to 5.21, *p*=0.63 and −1.08, 95% CI −4.91 to 2.75, *p*=0.58 respectively).

At six weeks, there was no evidence that premenopausal status affected sex differences in treatment discontinuation of citalopram and reboxetine (odds ratio 1.00, 95% CI: 0.37 to 2.73, *p*=1.00). Results were similar for treatment discontinuation at 12 weeks (odds ratio 1.07, 95% CI: −0.45 to 2.56, *p*=0.88). Repeating the same analysis on participants at or over the age of 45 years yielded similar results at six and 12 weeks of treatment (odds ratio 2.41, 95% CI 0.56 to 10.35, *p*=0.24 and 1.40, 95% CI 0.38 to 5.10, *p*=0.61 respectively).

## Discussion

### Summary of main findings

We tested the hypothesis that women would have greater reductions in depressive symptoms than men after treatment with a SSRI compared with a NaRI control. We also investigated whether women had a better depression severity prognosis after antidepressant treatment, regardless of antidepressant class. We found no evidence in support of either of these hypotheses.

This finding suggests that oestrogen driven serotonergic difference between women and men does not lead to an observable difference in the clinical effectiveness of SSRI treatment. The minimal clinically important difference (MCID; the smallest change in scores required for a patient to detect feeling better) in BDI-II scores is a reduction of 17.5% (Button et al., 2015). In our study, the MCID would therefore be a mean reduction in BDI-II scores at follow-up of six points. This means that even in the extreme of the confidence interval of the interaction term, whereby women on reboxetine fared worse than men on citalopram by 2.6 BDI points, the difference in effect would not be clinically meaningful. Our evidence is therefore that women and men have similar responses to citalopram compared with reboxetine.

We also examined sex differences in depression severity prognosis, irrespective of antidepressant class. We initially found evidence that women had higher depression severity scores than men at the six and 12 week follow-up time-points. However, after we adjusted for baseline severity of depressive symptoms, there was no evidence of a worse depression severity prognosis for women compared with men. This suggests that more severe depressive symptoms in women compared with men after antidepressant treatment can be attributed to higher depression severity prior to treatment.

We investigated sex differences in antidepressant tolerability. There was no evidence of any difference in tolerability between men and women. This suggests that women are unlikely to differentially tolerate SSRIs compared with men.

We found no evidence that menopausal status affected our findings. This suggests that hormonal fluctuations present in the perimenopausal and postmenopausal period are unlikely to affect efficacy of SSRI antidepressants in a clinically relevant way.

### Strengths and limitations

Our study had a large sample size (*N*=601) and low attrition rates which did not differ according to treatment allocation (there was 91% retention at six weeks and 81% at 12 weeks). There was a difference in adherence, with patients taking reboxetine have lower adherence to their medication compared with those taking citalopram. However, when we restricted analyses to participants who had adhered to their medication (reboxetine or citalopram), our results were unaltered. Allocated medications were prescribed at doses that are standard for UK primary care (http://bnf.org/bnf/index.htm). GPs retained clinical responsibility for patient care throughout the study and were free to increase the dose of allocated medication where appropriate. The dose of medication was increased for 20% of GENPOD participants. While it is difficult to recruit trial participants who are fully representative of patient populations, the exclusion criteria in GENPOD were kept minimal (inability to complete trial self-administered questionnaires, bipolar disorder, major substance or alcohol misuse disorders and current psychosis). However, it is important to note that many people who receive antidepressants in the general population do not meet diagnostic criteria for depression (Lewis et al., 2019). Our results may not generalize to people with less severe depression, although, in GENPOD, there was no evidence that severity of depression affected the treatment effect (Wiles et al., 2012). Citalopram has a similar pharmacological profile to other SSRIs and acts via similar mechanisms. We would therefore expect our results to apply to other SSRIs when used in this population.

Although it is possible that higher doses of antidepressants may have influenced the effects of sex on treatment response, the lower range of the licensed dose of SSRIs has been shown to achieve optimal balance between efficacy, tolerability and acceptability in the acute treatment of major depression (Furukawa et al., 2019). Larger doses of antidepressants incur a higher side-effect burden and it may be that any differential relationship between sex and high-dose antidepressants is not clinically significant.

There was no placebo control in the GENPOD trial and this may have affected our conclusions about effectiveness. However, our analysis does not focus on effectiveness as an outcome but instead investigates comparative sex differences in effectiveness of antidepressant classes, with reboxetine used as a comparator. The fact that GENPOD used a NaRI as a comparator would have improved our ability to detect potential differences in response to SSRIs between women and men, because NaRIs target different neurotransmitter systems from SSRIs.

Both the BDI and CIS-R scores used in GENPOD relied on patient self-reports. Additionally, part of the analysis was conducted on group change (i.e. women taking citalopram *vs*. women taking reboxetine, or premenopausal *vs*. peri- or postmenopausal women), and so was not affected by potential sex differences in self-reported symptoms. Using HADS depression scores to investigate sex differences on reduction in depression symptom severity yielded similar results.

Our analyses on the effect of postmenopausal and premenopausal status were conducted using age cut-offs based on average ages of menopause and perimenopause in the UK. Exact data on individual menopausal status including hormone panels may have improved our ability to test any effects of menopausal status on treatment response and tolerability.

Our sample was mostly White – 96.0% of women and 95.0% of men were White and this is not representative of people with depression in the UK. However, we are not aware of any evidence to suggest an effect of ethnicity on antidepressant outcomes.

### Comparison with existing studies

Our findings are consistent with several other large trials and cohort studies which did not find evidence that women responded more favourably to SSRIs than men. Our study extends these studies by employing a large randomized design and using a NaRI antidepressant as a control.

Our findings are consistent with other studies not finding any evidence of a differential effect of menopausal status on SSRI treatment effectiveness. Our study has not found evidence to suggest sex differences in antidepressant tolerability between men and women. Our study extends previous studies by using a NaRI control, employing a prospective design with minimal exclusion criteria, measuring treatment adherence and tolerability and investigating effects of menopausal status on treatment response and tolerability.

### Implications of our findings

The lack of clinical translation of the oestrogen-driven serotonergic effects to antidepressant response and tolerability may be due to the way in which SSRIs bring about antidepressant effects. In brief, increased serotonin availability activates a complex pathway downstream of the serotonin receptor, which results in increased gene transcription and, eventually, neurogenesis. The pathway has multiple components and an increase in serotonin availability does not guarantee amplification of downstream components which may become saturated. It may be that maximal serotonin transporter blockade brought about by SSRIs at clinical doses is potent enough to produce a pharmacological effect on serotonin availability which surpasses differences in serotonergic circuitry and transporter regulation between women and men.

We did not find evidence of an effect of sex on depression severity outcomes with an SSRI compared with a NaRI antidepressant. Our study also demonstrates that differences in depression severity according to sex after antidepressant therapy can be attributed to higher severity of depressive symptoms in women at baseline. It is therefore unlikely that clinicians will find therapeutic benefit in tailoring their antidepressant prescribing according to sex. We found no evidence of a relationship between menopausal status and effectiveness or tolerability of SSRI or NaRI antidepressants. Tailoring treatment to menopausal status in clinical practice may not be advantageous. We also did not find any evidence of an effect of sex or menopausal status on antidepressant tolerability with SSRI or NaRI antidepressants, suggesting that these two classes are equally tolerated by men and women.

## Supplemental Material

sj-pdf-1-jop-10.1177_0269881120986417 – Supplemental material for Sex differences in depressive symptoms and tolerability after treatment with selective serotonin reuptake inhibitor antidepressants: Secondary analyses of the GENPOD trial Supplemental material, sj-pdf-1-jop-10.1177_0269881120986417 for Sex differences in depressive symptoms and tolerability after treatment with selective serotonin reuptake inhibitor antidepressants: Secondary analyses of the GENPOD trial by Marilia Gougoulaki, Glyn Lewis, David J Nutt, Tim J Peters, Nicola J Wiles and Gemma Lewis in Journal of Psychopharmacology
