# Supplementary material for: Sex differences in depressive symptoms and tolerability after treatment with selective serotonin reuptake inhibitor antidepressants: Secondary analyses of the GENPOD trial
Source: J Psychopharmacol. 2021 Feb 26;35(8):919–27. doi: 10.1177/0269881120986417 (PMC8358567; doi:10.1177/0269881120986417)
Supplement: sj-pdf-1-jop-10.1177_0269881120986417 – Supplemental material for Sex differences in depressive symptoms and tolerability after treatment with selective serotonin reuptake inhibitor antidepressants: Secondary analyses of the GENPOD trial [file sj-pdf-1-jop-10.1177_0269881120986417.pdf]

Supplementary Table 1: Means and adjusted differences in mean (95% CIs) BDI-II scores at 6 and 12-weeks, according to citalopram and reboxetine groups in women and men likely to be premenopausal (age <45).

**6 weeks (n=374)**					
**Sex**	**Citalopram**		**Reboxetine**		**Adjusted difference (95% CI) between citalopram and reboxetine**
	**n**	**Mean (SD)**	**n**	**Mean (SD)**	
**Women**	136	17.9 (10.4)	132	21.3 (11.3)	3.54 (0.98 to 6.10)
**Men**	53	16.8 (10.2)	53	17.0 (10.3)	1.33 (-2.51 to 5.18)
**12 weeks (n=326)**					
**Sex**	**Citalopram**		**Reboxetine**		**Adjusted difference (95% CI) between citalopram and reboxetine**
	**n**	**Mean (SD)**	**n**	**Mean (SD)**	
**Women**	126	14.9 (11.8)	115	15.7 (12.2)	1.06 (-1.83 to 3.95)
**Men**	45	13.3 (8.8)	40	13.7 (10.2)	1.76 (-2.23 to 5.76)

Differences in means are calculated from linear regression models, with higher scores indicating a worse outcome on reboxetine compared with citalopram (the reference category).

Differences in means are adjusted for the stratification variable (depression symptom severity <28 or >=28 on the CIS-R), centre and continuous baseline BDI-II scores

Supplementary Table 2: Means and adjusted differences in mean (95% CIs) BDI-II scores at 6 and 12-weeks, according to citalopram and reboxetine groups in women and men likely to be peri- or postmenopausal (age >=45).

**6 weeks (n=172)**					
**Sex**	**Citalopram**		**Reboxetine**		**Adjusted difference (95% CI) between citalopram and reboxetine**
	**n**	**Mean (SD)**	**n**	**Mean (SD)**	
**Women**	49	23.8(11.9)	56	18.1 (9.9)	-4.01 (-7.90 to -0.14)
**Men**	36	18.8 (10.4)	31	19.4 (10.3)	-2.17 (-7.00 to 2.66)
12 **weeks (n=160)**					
**Sex**	**Citalopram**		**Reboxetine**		**Adjusted difference (95% CI) between citalopram and reboxetine**
	**n**	**Mean (SD)**	**n**	**Mean (SD)**	
**Women**	48	19.9 (12.5)	51	15.5 (10.0)	-2.70 (-7.07 to 1.73)
**Men**	34	13.6(10.5)	27	13.7 (11.4)	-2.11 (-7.74 to 3.51)

Differences in means are calculated from linear regression models, with higher scores indicating a worse outcome on reboxetine compared with citalopram (the reference category).

Differences in means are adjusted for the stratification variable (depression symptom severity <28 or >=28 on the CIS-R), centre and continuous baseline BDI-II scores

Supplementary Table 3: Means and adjusted differences in mean (95% CIs) HADS scores at 6 and 12-weeks, according to citalopram and reboxetine groups in women and men

**6 weeks (n=546)**					
**Sex**	**Citalopram**		**Reboxetine**		**Adjusted difference (95% CI) between citalopram and reboxetine**
	**n**	**Mean (sd)**	**n**	**Mean (sd)**	
**Women**	185	7.97 (4.86)	188	8.47 (4.93)	0.65 (-0.29 to 1.60)
**Men**	89	7.64(5.04)	84	7.72 (4.96)	0.37 (-1.00 to 1.74)
**12 weeks (n=486)**					
**Sex**	**Citalopram**		**Reboxetine**		**Adjusted difference (95% CI) between citalopram and reboxetine**
	**n**	**Mean (sd)**	**n**	**Mean (sd)**	
**Women**	174	6.98 (5.11)	166	6.61 (4.78)	-0.26 (-1.31 to 0.78)
**Men**	79	6.06 (4.67)	67	6.37 (4.68)	0.59 (-0.89 to 2.08)

Differences in means are calculated from linear regression models, with higher scores indicating a worse outcome on reboxetine compared with citalopram (the reference category).

Differences in means are adjusted for the stratification variable (depression symptom severity <28 or >=28 on the CIS-R), centre and continuous baseline HADS scores

Supplementary Table 4: Odds ratios (ORs) and 95% CI for treatment discontinuation in women and men taking citalopram and reboxetine at 6 and 12 weeks

		**OR**	**95% CI**	**P value**
**6 weeks**	**Female, reboxetine**	2.43	1.53 to 3.88	<0.001
	**Male, reboxetine**	1.36	0.60 to 3.06	0.47
	**Male, citalopram**	0.94	0.49 to 1.80	0.85
**12 weeks**	**Female, reboxetine**	2.40	1.60 to 3.61	<0.001
	**Male, reboxetine**	1.18	0.58 to 2.41	0.65
	**Male, citalopram**	1.12	0.66 to 1.88	0.68
Reference variable: Female, citalopram				
